# Consumer acceptance of using a digital technology to manage postpartum depression

**DOI:** 10.3389/fgwh.2022.844172

**Published:** 2022-08-25

**Authors:** Jian Jenny Tang, Indira Malladi, Melva T. Covington, Eliza Ng, Shailja Dixit, Sid Shankar, Stan Kachnowski

**Affiliations:** ^1^The Mount Sinai Hospital, Obstetrics, Gynecology and Reproductive Science, New York, NY, United States; ^2^Curio Digital Therapeutics, Princeton, NJ, United States; ^3^Coalition for Asian-American Independent Physician Associations, New York, NY, United States; ^4^Health Innovation and Technology Laboratory, New York, NY, United States

**Keywords:** postpartum depression, digital therapeutics, human factors, OBGYN, Cognitive Behavioral Therapy, Interpersonal Therapy, digital applications, behavioral health management

## Abstract

The goal of the study was to evaluate the end user experience using the MamaLift Plus app for 2 weeks to support the treatment of their postpartum depression (PPD). A total of 14 participants completed the study and their experiences are reported in this publication. Participants reported that MamaLift Plus is an acceptable, highly usable, and practical mobile tool to use weekly for the management of their PPD. More research is warranted to evaluate the benefit of digital behavior health interventions, especially in patient populations where mental health care may be limited or harder to access by patients.

## Introduction

Digital health is transforming the health care delivery system, particularly in the way that patients and providers are engaging to make health care decisions (1). Patients are now more empowered to use technologies to seek services, understand their health condition, and jointly make decisions with their healthcare providers. This kind of engagement can lead to improvements in health outcomes. The objective of this study was to evaluate the consumer behavioral health experience using a digital platform. This study focused specifically on assessing the feasibility, usability, and acceptability of the MamaLift Plus digital therapeutic in addressing issues associated with the management of PPD after childbirth.

## Background

### Postpartum depression

PPD is a debilitating condition that affects about one in seven mothers following childbirth (2). Symptoms of PPD include sadness, anxiety, wide mood swings, guilt, sense of worthlessness, helplessness, irritability or restlessness, loss of interest, energy, problems concentrating, difficulty falling asleep or sleeping too much, and overeating or loss of appetite (3). Women with severe PPD may even exhibit thoughts of harming herself or her loved ones, including her baby. In fact, recent studies point to a trend that suggests death by self-harm through suicide or overdose has become a leading cause of death for women in the perinatal period (4). Other studies estimated that suicide accounts for about 20% of postpartum deaths and is the second most common cause of mortality in postpartum women (2, 5).

Perinatal mental disorders, like PPD, may also have devastating transgenerational effects (6). Poor maternal mental health can have a devastating and long-lasting impact on the heath of a child. Children of mothers with perinatal depression may exhibit poor growth development, insecure attachment, elevated rates of illness, and emotional and behavioral problems. Moreover, studies suggest that cognitive development is compromised in four-year-old children of mothers who had postpartum depression. It has been suggested that impaired patterns of interaction between mothers and infants may be an important determinant of some adverse child outcomes (7).

Studies have also investigated the effect of PPD symptoms on partners and suggest that relationships can be negatively affected. Partners of affected women experienced greater levels of their own stress, anxiety, and depression compared with partners of women without PPD symptoms (8). One study of 626 children and their families examined the influence of fathers' characteristics on the development of their child when exposed to maternal postpartum depression. Paternal workforce participation during the first 2 years of a child's life can also have an impact on the behavioral outcomes associated with anxiety, hyperactivity, and aggression. This can also predict subsequent child development over the next 10 years (9).

### Current clinical practice patterns

According to practice guidelines issued by the American College of Obstetricians and Gynecologists (ACOG) in the United States (U.S.), obstetric care providers should screen patients at least once during the perinatal period for depression and anxiety symptoms using a standardized, validated tool (10). In practice in the U.S, women are typically screened for postpartum depression during the first postnatal visit, typically 2–6 weeks after delivery. Those who have a positive screen may undergo a variety of clinical assessments to determine or confirm their PPD diagnosis. According to the ACOG guidelines, providers should develop a postpartum mental health care plan for women with PPD based on the level of risks and severity of their symptoms. The care plan should include anticipatory guidance regarding signs and symptoms of perinatal depression or anxiety and management recommendations for women with anxiety, depression, or other psychiatric issues identified during pregnancy or in the postpartum period (11). Research suggests that Cognitive Behavioral Therapy (CBT) should be the first line of treatment for postpartum depression (12). Despite this, evidence suggests that there are variations in the adoption of screening guidelines. Specifically, 20% of women (1 in 5) are not asked about depression symptoms during prenatal visits even though it is standard assessment for care and prevention (13). Most women are not properly diagnosed and treated due to lack of adherence to clinical screening guidelines, limited access to behavioral health services, lack of training in the management of mental health conditions, and personal stigma (14–18). Over half of pregnant women with depression symptoms were not being treated (13).

To gain a better understanding of current practice patterns, we conducted a Delphi Panel (unpublished data) to gain a better understanding of unmet need and clinical practice. The Delphi Panel uses a scientific methodology and a structured approach to collect knowledge and achieve consensus. Our Delphi panel was conducted through a series of questionnaires with structured answers to allow for quantitative analysis and qualitative information gathering. Panelists held varied opinions of the ideal time for PPD screening. Most of the panel experts reported that they themselves conduct additional assessments if they suspect that PPD is present, as opposed to using a behavioral health provider. However, when a patient is identified as having PPD, almost all the panelists said that they refer the patient to a specialist for subsequent management. The recommended duration of intervention for PPD is 3–6 months, with panelists indicating a strong preference for a maintenance program after the active treatment period. All panelists stressed the importance of discussing and addressing physiological and biological changes associated with PPD. Panelists also suggested that approaches to increase adoptability of new technologies should include destigmatizing mental health, providing mental health education, improving access to care, and encouraging family social support.

### Psychotherapy for PPD

Evidence indicates that psychotherapy in the form of Cognitive Behavioral Therapy (CBT), Behavioral Activation Therapy (BAT), Dialectical Behavior Therapy (DBT), and Interpersonal Therapy (IPT) are effective in the treatment of postpartum depression (19–28). CBT helps people to arrive at a more realistic view of the world by teaching them to recognize their inaccuracies in thinking (29). IPT is a time-limited form of psychotherapy that makes a practical link between a person's mood and life events that either trigger or follow from the onset of the mood disorder (30). BAT teaches people to re-engage in their lives through focused activation strategies. These strategies counter patterns of avoidance, withdrawal, and inactivity that may exacerbate depressive episodes (31). DBT is based on treatment strategies that help a person develop greater acceptance of oneself, of others, and of life in general by drawing primarily on principles of meditation and mindfulness (32). In patients who are at risk of suicide or suicidal ideation, referrals to psychiatric care and/or hospital facilities may be necessary. Optimal treatment for patients in this context is the initiation of psychotherapy. If optimal results are not achieved, then medication is the next line of recommended treatment (11).

### Mobile applications as a solution to address needs and gaps of current clinical practice

Despite clear guidelines that recommend screening and inclusion of a postpartum mental health care management plan of cognitive-behavioral therapy for at-risk women of PPD, the evidence indicates that nearly half of pregnant women with symptoms of depression remain untreated (13). There are various factors that may contribute to this disparity, including a lack of adherence to clinical screening guidelines, lack of training in the management of mental health conditions, and limited availability of resources. These factors, combined with the personal stigma and limited access to behavioral health services reported by women at-risk in the perinatal period, contribute to the staggering proportions of women who are undiagnosed and underdiagnosed with PPD.

Mobile-based platforms can offer providers and patients with a novel opportunity to address the gaps in adherence to practice guidelines and clinical management for improvements in patient outcomes. Smartphone-enabled mobile applications have been shown to be effective tools for identifying and supporting the management of at-risk patients (33). Mobile applications can be effective in screening for PPD and achieve a high level of sensitivity and accuracy. These applications may also have utility for mental health interventions in that they are quick to perform, have low threshold access, and demonstrate cost-effectiveness (34). Moreover, mobile-based technologies may require limited training by healthcare providers and can be deployed at the point of care for the management of mental health conditions. By leveraging features, such as natural language processing and artificial intelligence, mobile-based technologies can provide accelerated and actionable alerts for behavioral health management (35).

By engaging with a self-guided, mobile-based intervention, new mothers do not need to fear the stigma of mental health. Moreover, these tools offer privacy to enable patients to engage in behavior health management without the fear of being judged or shamed. Moreover, digital health technologies may reduce social stigma by validating a women's experiences and normalizing symptoms in the perinatal period (36). The wide diffusion of smartphones across all sectors of society has enabled the opportunity to reach more people who need access to behavioral health services, especially women with limited resources and access to providers who can provide help for postpartum depression, including non-white, Hispanic, urban-dwelling, and rural-dwelling women in the U.S. (37, 38).

Reports demonstrate that 85% of Americans report owning a smartphone, including women who self- report as being white, black (or African-American), or Hispanic report. Among rural-dwellers in the U.S., smartphone ownership is estimated to be similarly high or at least 80% (39). Approximately 80% of smartphone users use their phone to research conditions or access health information. Some studies indicate that Black and Latino women are more likely to use smartphones to research health conditions than White women who use smartphones for the same purpose (40).

While there has been sufficient evidence that demonstrates barriers to receiving CBT in the perinatal period, the acceptability and efficacy of digital health interventions is less well known (41–43). Effective use of mobile technologies studies note that user engagement or use of digital health interventions or applications by women in the perinatal period remains sub-optimal. During this period, women may experience personal barriers to receiving adequate health care and limited engagement with digital health interventions due to factors such as lack of motivation, lack of cultural competence by providers, limits in health literacy or competing childcare or family priorities (21, 44).

### Development of MamaLift plus digital intervention for PPD

The MamaLift Plus digital intervention was based on a robust process of evidence-based research principles, clinical practice experience, and patient outcomes from a legacy prototype (“Be-A-Mom”) that was developed in Europe by the University of Coimbra. Be-A-Mom is a web-based intervention developed initially to address women at-risk for PPD, but was subsequently expanded to offer treatment to women with a confirmed diagnosis of PPD. We leveraged the learnings and understanding from Be-A-Mom to further refined the content and structure to develop MamaLift Plus presented in this analysis.

Early in the content development stage, we conducted an extensive literature review and focus group with mental health professionals (as mentioned earlier). We examined the most relevant characteristics, goals, and content of interventions designed to treat PPD. In the research (45), a scoping review was performed to identify the therapeutic goals included in preventive interventions for PPD and to identify the intervention characteristics that may prompt the effectiveness of approaches used for PPD. The review confirmed the following.

CBT and IPT, both short-term therapies, were considered effective in the prevention and treatment of PPD (22, 46, 47). In addition, there is evidence that CBT for PPD should include perinatal-specific concerns (e.g., culturally endorsed beliefs about motherhood, the impact of pregnancy and of a new infant on a woman's identity, and the ability to sustain and engage in previously valued and meaningful activities) and interpersonal domains (e.g., improving appropriate social support) (48). Recent developments in CBT treatment for depression have also highlighted the role of third-wave CBT approaches, such as Acceptance and Commitment Therapy and Compassion- Focused Therapy (49, 50). The need for effective and timely interventions is critical to address the growing impact of PPD on the lives of patients. The MamaLift Plus mobile application, developed by Curio^TM^ Digital Therapeutics, Inc., is a digital therapeutic designed to support the treatment of patients with mild-to-moderate PPD. It is based on the evidence-based principles of CBT, IPT, DBT, and BAT and incorporates specific themes and content to be deployed in the perinatal period of pregnancy. MamaLift Plus patients spend 10–15 min per day in this self-guided program to obtain the optimal therapeutic benefit. Content is offered in a variety of modalities, including video, audio, animations, and text. All content has been adapted to reflect the diversity of its intended audience. MamaLift Plus can be used as a standalone therapy or in conjunction with direct medical care provided by a behavioral health therapist. MamaLift Plus engages patients through a variety of interactive exercises and personalized feedback mechanisms presented in self-guided modules. Moreover, it includes gamification features developed to enhance patient engagement and reduce attrition.

## Study objectives

The objective of the study was to conduct a Human Factors Trial (HFT) with MamaLift Plus application in the U.S. population with the goal of providing insights into tool design and usability. The information collected is intended to inform the future development of the digital intervention and improve user experience and acceptance.

### Study design

The design of this study was to evaluate the user experience (UX), including feasibility, usability, and acceptability of the MamaLift Plus program among participants with PPD for 2 weeks (or to provide behavior health intervention modules that were estimated to take 2 weeks to complete). This was a single-arm, mixed-methods (quantitative and qualitative) study with a total of 14 participants.

## Methods

### Recruitment and study participants

Eligibility criteria for participation by women in this study were: (a) able to read, write, and speak English and provide written informed consent prior to enrollment; (b) between 18 and 50 years of age; (c) confirmed to have given live birth within the last 4 months prior to the start of the study; (d) diagnosed with mild to moderate PPD within the last 4 months or having an EPDS score between nine and 21 during screening; (e) “0/Never” or “1/Hardly Ever” engaged in self-harm on the EPDS (question 10); (f) willing to use a mobile app and owning an iOS enabled mobile phone; (g) able to access wireless internet connectivity in the home and being willing to connect devices to their home Wi-Fi network; (h) not diagnosed with any Serious Mental Illnesses (SMIs).

Participants in the postpartum period were recruited online between April 2021 and May 2021. The initial recruitment strategy centered around advertising using a digital flier on social media sites (e.g., LinkedIn) and to on-line groups focused on women's health technology and digital therapeutics. Digital flyers were also posted on Facebook, Twitter, and Instagram. The protracted flow of participants who met all inclusion criteria inspired a change in eligibility requirements and recruitment strategies. Original eligibility criteria disqualified individuals who answered anything other than “0/Never” to the self-harm question on the EPDS (question 10). By opening eligibility criteria to include those who answer “1/Hardly Ever” to the self-harm question in May 2021, the flow of participants was improved. Additionally, the recruitment strategy was modified to include advertising on Reddit social media platform, which resulted in a rapid increase in the identification and enrollment of interested participants (from 13 to 56). In all, 14 participants were fully enrolled, and one patient dropped out during the course of the study.

### Measures

Data sources included online questionnaire data, semi-structured interviews, and device data. The end-line questionnaires included the System Usability Scale (SUS), Acceptability questionnaire, User Experience Questionnaire, and Feasibility Questionnaire. Full questionnaires may refer to the digital application by its older name, Stella-t. The name of the digital application has since been changed to MamaLift Plus.

Device usage was measured by participant use of MamaLift Plus within a given time period and utilization of its tracking features that were built into the application. These trackers included a mood tracker, sleep tracker, and activity tracker. Acceptability of device was assessed using an end-line acceptability questionnaire (SUS). Feasibility was assessed by asking women about the practicability of spending time on the MamaLift Plus application every day and User Experience on the app was measured using a set of exploratory questions.

### Study procedures

Participants were expected to spend at least 10 to 15 min per day using MamaLift Plus for 2 weeks. It was recommended that participants spend that time completing the psychotherapeutic lessons delivered through the “Daily Learning” feature of the MamaLift Plus mobile application. In addition, women were encouraged to track mood, sleep, and activities using the trackers at least once per day. Participants were also encouraged to complete one psychotherapeutic lesson per day for 14 calendar days (or 2 weeks) that amounted to a total of 14 Daily Learning sessions. However, women were allotted up to 5 weeks to complete the lessons to accommodate their schedules, in consideration of the unpredictable nature of being a new mother.

## Results

### Acceptability

**Figure 1:**
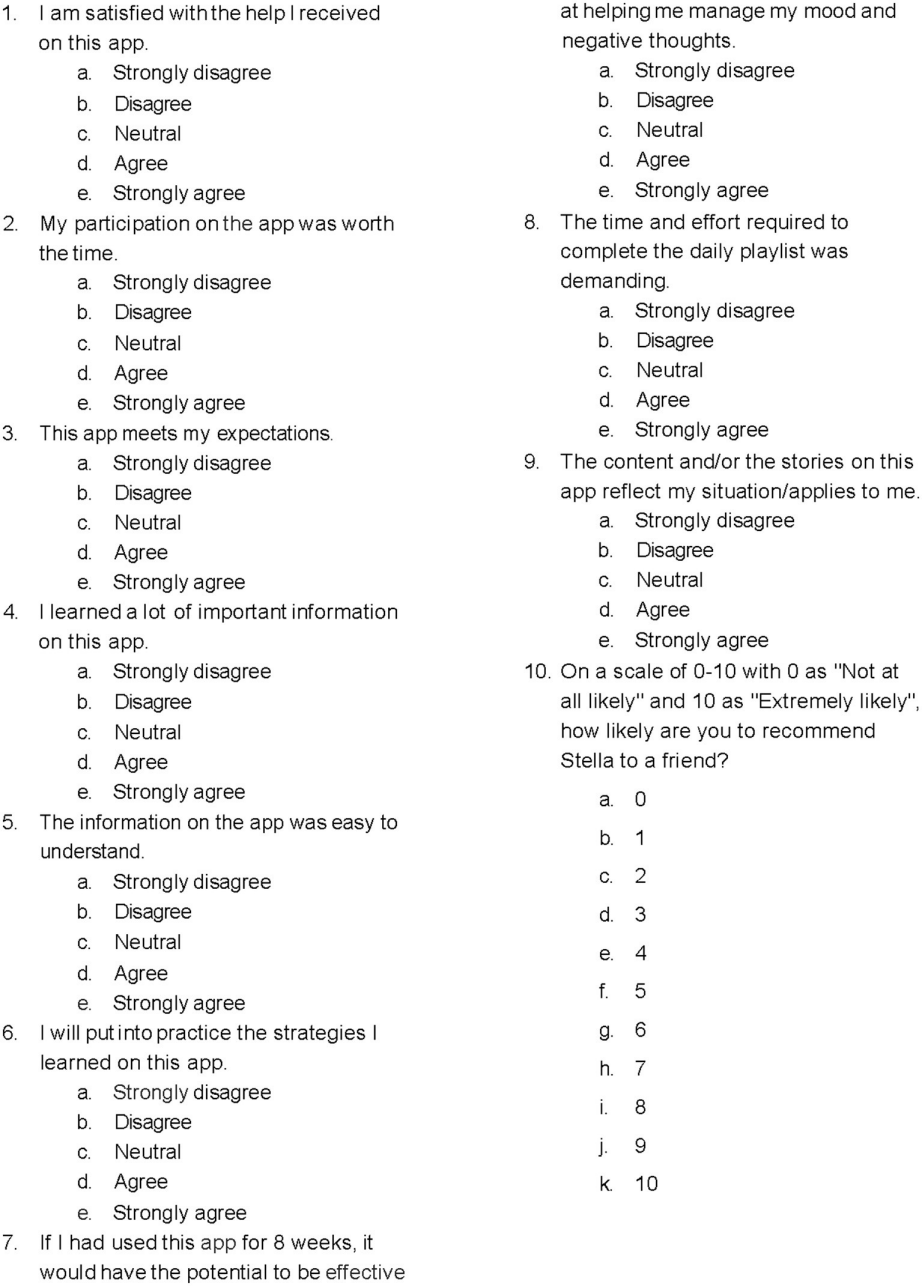
Acceptability questionnaire.

**Figure 2:**
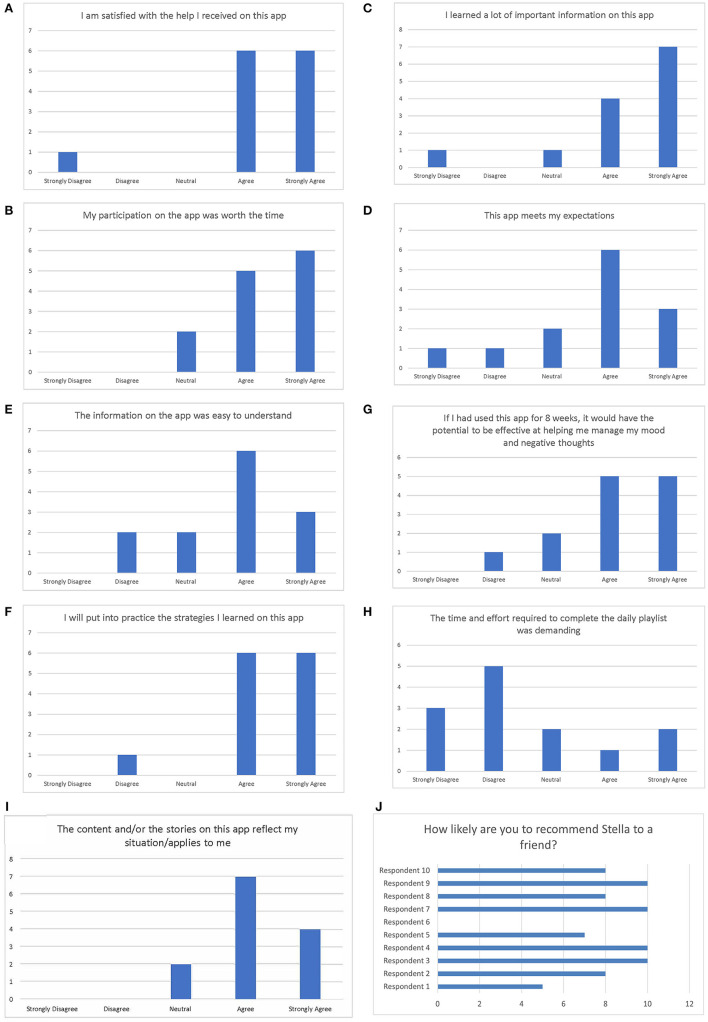
**(A–J)** Results of the acceptability questionnaire.

The results from the end line acceptability questionnaire (see Figure 1) indicate that the MamaLift Plus app is an acceptable tool as reported by participants in the study. When asked if they were satisfied with the help received on this app, 6 of 13 (46.2%) participants strongly agreed and 6 of 13 (46.2%) agreed (Figure 2). When asked if their participation on the app was worth the time, 46.2% of participants strongly agreed and 38.5% agreed that the app was worth their time. When asked if they learned a lot of important information on this app, 53.8% of participants strongly agreed and 30.8% agreed. When asked if this app meets their expectations, 23.1% strongly agreed and 46.2% agreed. Twenty-three percent and 46.2% of participants strongly agreed and agreed, respectively, to the statement, “The information on the app was easy to understand.” More than half of participants either strongly agreed or agreed (46.2 and 46.2%, respectively) to the acceptability statement, “I will put into practice the strategies that I learned on this app.”

About 38% of participants both strongly agreed and the same proportion agreed to the statement, “If I had used this app for 8 weeks, it would have the potential to be effective at helping me manage my mood and negative thoughts.” Over 23 and 38.5% strongly disagreed and disagreed, respectively, to the statement, “The time and effort required to complete the daily playlist was demanding.” More than 30 and 53% strongly agreed and agreed, respectively, to the statement, “The content and/or stories on this app reflect my situation/applies to me.”

A final tenth question was added to the Acceptability questionnaire mid-way through the study to calculate a Net Promoter Score (NPS). Ten out of 13 participants had the opportunity to respond to the question, “On a scale of 0–10 with 0 as “Not at all likely” and 10 as “Extremely likely,” how likely are you to recommend Stella-t to a friend?” Four out of 10 (40%) participants were promoters, another four were passive (40%), and two out of 10 (20%) were detractors (Figure 2). The NPS of MamaLift Plus was calculated to be 20.

### Usability

**Figure 3:**
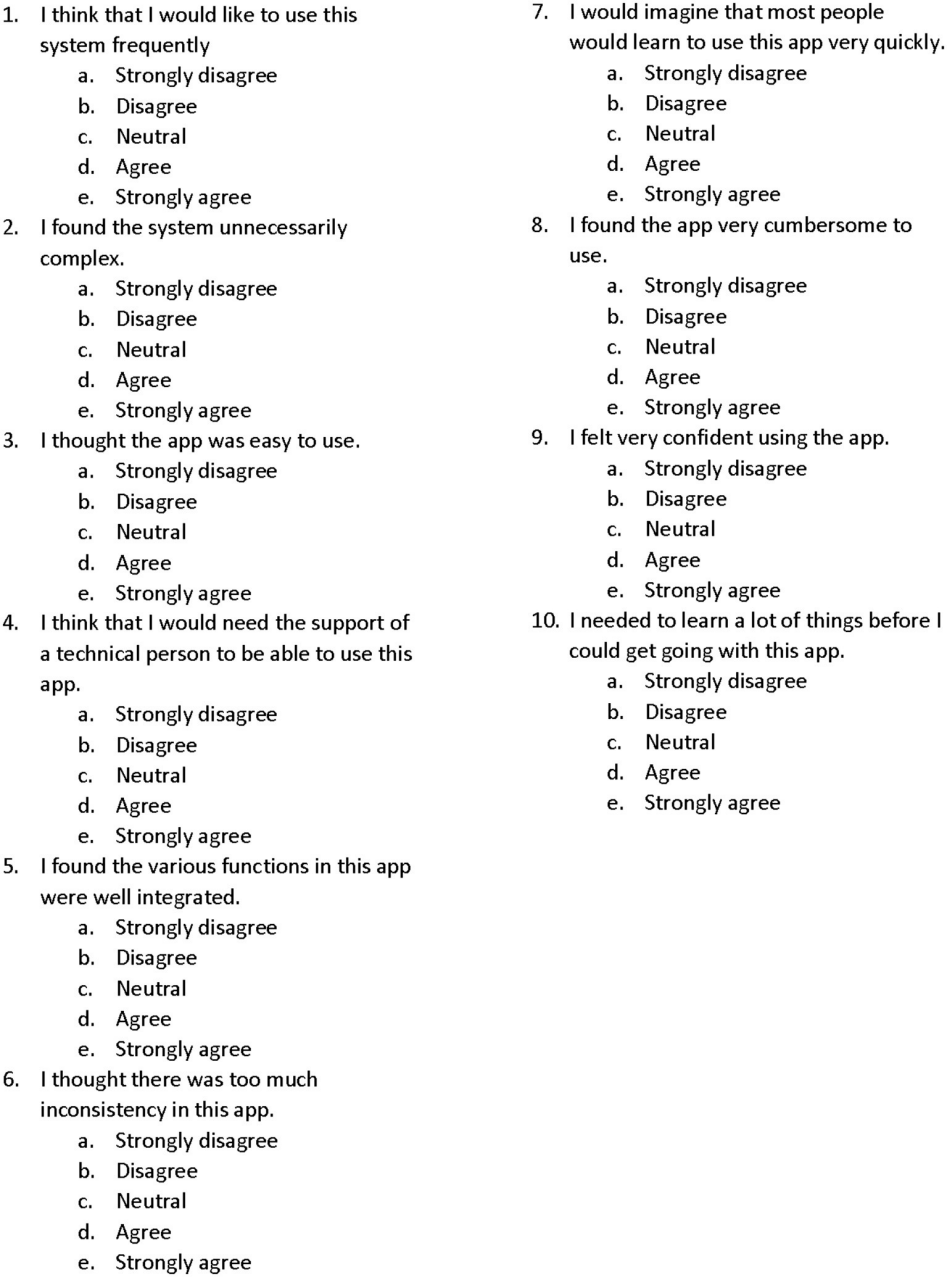
Systems usability scale questionnaire.

The results of the SUS Questionnaire (Figure 3) indicated that the MamaLift Plus app has high usability among the target population, with 30.8% of participants strongly agreed and 46.3% agreed that they would use the device frequently. Nearly half (46.2%) of participants strongly agreed and 23.1% agreed that the devise was easy to use. When asked if they found the various functions in this device to be well integrated, 23.1% strongly agreed and 53.8% agreed when asked whether they thought most people would learn to use this device very quickly, 38.5% strongly agreed and 46.2% agreed. When asked if they felt confident using this device, 46.2% of participants strongly agreed and 23.1% agreed (Table 1). Over a third (38.5%) of participants and 30.8% of participants would strongly disagree and disagree, respectively, to the statement, “I found the device unnecessarily complex.” Conversely, 61.8% and 30.8% strongly disagreed and disagreed to the statement, “I would need the support of a technical person to be able to use this device.”

**Table 1:** Results from the human factors trial usability questionnaire.

**Positive assessment statements**	**Response options**	**I would like to use this device frequently**	**I thought the device was easy to use**	**I found the various functions in this device were well integrated**	**I would imagine that most people would learn to use this device very quickly**	**I felt very confident using the device**
	Strongly agree	30.8%	46.2%	23.1%	38.5%	46.2%
	Agree	46.3%	23.1%	53.8%	46.2%	23.1%
	Neutral	0%	30.8%	7.7%	7.7%	30.8%
	Disagree	15.4%	0%	15.4%	7.7%	0%
	Strongly disagree	7.7%	0%	0%	0%	0%
**Negative Assessment Statements**	**Response Options**	**I found the device unnecessarily complex**	**I would need the support of a technical person to be able to use this device**	**I thought there was too much inconsistency in this device**	**I found the device very cumbersome to use**	**I needed to learn a lot of things before I could get going with this device**
	Strongly agree	0%	0%	7.7%	7.7%	15.4%
	Agree	15.4%	7.7%	7.7%	15.4%	0%
	Neutral	15.4%	0%	30.8%	15.4%	7.7%
	Disagree	30.8%	30.8%	30.8%	38.5%	46.2%
	Strongly disagree	38.5%	61.8%	23.1%	23.1%	30.8%

A total of 23.1% of participants strongly disagreed to the statement, “I thought there was too much inconsistency in this device,” while 30.8% disagreed and 30.8% remained neutral. Also, 23.1% of participants strongly disagreed, and 38.5% of participants disagreed, to the statement, “I found the device very cumbersome to use.” When asked if they needed to learn a lot of things before they could get going with this device, 30.8% of participants strongly disagreed and 46.2% disagreed (Table 1). The average SUS score was 72.5, with a mode of 50 and median of 77.5.

### Exploratory questions

**Figure 4:**
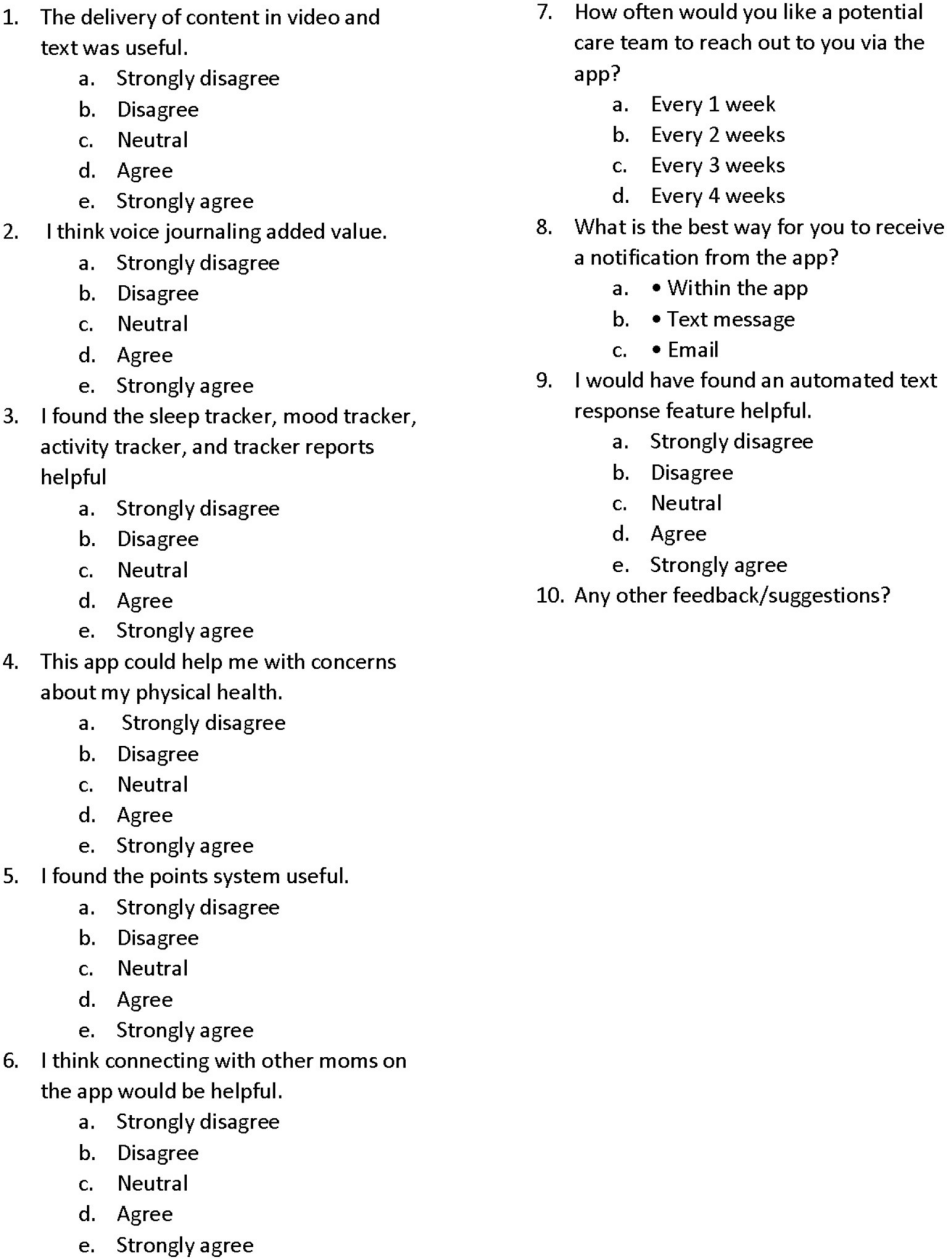
User experience questionnaire.

**Figure 5:**
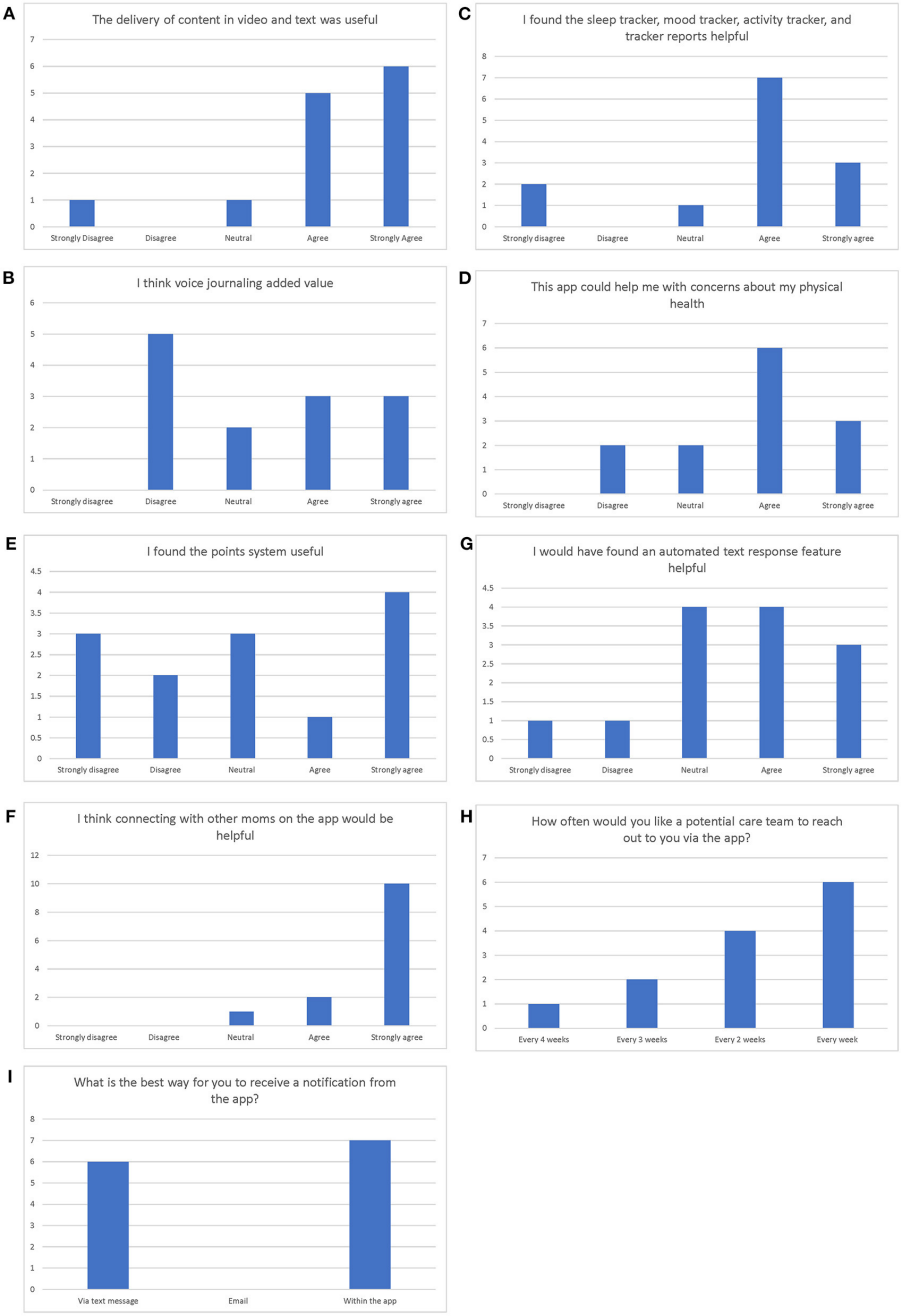
**(A–I)** Results of the user experience questionnaire.

The user experience of the app as measured by exploratory questions (Figure 4) was generally positive (Figure 5). The delivery of content in video and text was useful (46.2% strongly agreed, 38.5% agreed). Interestingly, views on voice journaling were varied, with 23.1% indicating that they strongly agreed, 23.1% agreed, 15.4% were neutral, and 38.5% disagreed that voice journaling added value. Trackers were found to be helpful and 23.1% strongly agreed and 53.8% agreed to the statement, “I found the sleep tracker, mood tracker, activity tracker, and tracker reports helpful.” Participants indicated that this app could also help them with their physical health concerns, in which 23.1% strongly agreed and 46.2% agreed to this concept.

Views on the utility of the points system were also varied. Since points were not tied to any redemption engine during the HFT, the results confirmed the variability of opinions pertaining to this point system. In specific, nearly a third (30.8%) of the participants strongly agreed that the point system was useful, while 7.7% agreed, 23.1% remained neutral, 15.4% disagreed, and 23.1% strongly disagreed. However, participants overwhelmingly supported the concept of social support as indicated by the results of the statement, “I think connecting with other moms on the app would be helpful” (with 76.9% strongly agreed, 15.4% agreed).

Views on automated text response features were diverse; with 23.1% indicating that they strongly agreed that automated texts were helpful while 30.8% agreed, 30.8% remained neutral, 7.7% disagreed, and 7.7% strongly disagreed. Regarding the frequency with which they would like a potential care team to reach out to them *via* the app, 7.7% indicated a preference for every 4 weeks, 15.4% preferred every 3 weeks, 30.1% preferred every 2 weeks, and 46.2% preferred every week When asked about the best way for them to receive a notification from the app, 46.2% of participants preferred text message and 53.8% preferred in-app messages.

### Device usage

Device utilization indicated that 13 out of 14 (93%) enrolled participants who used the device completed the study. One participant dropped out during the course of the study and declined to provide feedback or experience data. Of the 13 participants who completed the study, 10 (77%) completed all 14 psychotherapeutic lessons in the program. Of the remaining 3 participants, 2 were incorrectly offboarded early and 1 person exceeded the maximum 5-week time period allotted to complete the psychotherapeutic lessons. The 10 patients who completed all psychotherapeutic lessons took a minimum of 14 days and maximum of 26 days to complete the lessons (Table 2). A majority (7 out of 10 participants), completed the lessons within 3 weeks and the average number of days spent completing all 14 psychotherapeutic lessons was 19 days.

**Table 2:** Device data usage.

**Participant number**	**No. of Mood tracker entries**	**No. of Sleep tracker entries**	**No. of activity tracker entries**	**Learning lessons completed** **(Total: 14 lessons)**	**Days to complete**
Participant 1	19	19	65	14	20
Participant 2	14	14	25	10	14
Participant 3	14	13	45	9	14
Participant 4	13	9	36	14	14
Participant 5	20	18	75	14	18
Participant 6	19	18	154	14	17
Participant 7	28	22	126	14	19
Participant 8	18	15	79	14	17
Participant 9	13	22	50	14	26
Participant 10	17	15	189	14	16
Participant 11	14	13	13	14	25
Participant 12	15	15	48	14	19
Participant 13	8	5	5	9	22

Device usage based on the counts of entries into the MamaLift Plus trackers for mood, sleep and activity level indicated high levels of engagement by participants. The number of entries into the mood tracker ranged from 8 to 28, with an average of 16 entries. The number of sleep tracker entries ranged from 5 to 22, with an average of 15 entries. The number of activity tracker entries ranged from 5 to 189, with an average of 70 entries (Table 2). Notably, 12 out of 14 participants were diligent about entering data into their sleep tracker on a daily basis (except for participants 4 and 13). This indicates that users largely found the sleep tracker to be acceptable.

There was little use of the journal entry feature of the MamaLift Plus mobile device that would have provided more qualitative feedback on the user experience. However, there were no differences in user behavior identified based on results of self-report questionnaires.

## Discussion

The early results of the Delphi Panel validate observations that there is variation in practice pattern in the timing of screening for PPD. The Delphi panel also confirmed the importance and challenges of access and use of mental health services. This is likely due to lack of available behavioral health providers and stigma of mental depression. The panel results confirm the potential ability of a digital tool to improve frequency of screening to enhance evaluation of depression and anxiety symptoms in the perinatal period using behavioral health therapy techniques by way of a mobile device.

The results of this study indicate that the MamaLift Plus digital application is acceptable, usable, and feasible for new mothers to address symptoms associated with PPD. The learnings extracted from this study will be leveraged to inform future enhancements to the application, especially around engagement. Participants indicated that use of the MamaLift Plus application for 10–15 min per day as suggested required to complete the daily playlist was too demanding. This was also consistent with feedback from participants on the feasibility questionnaire. The feedback also suggests that making the psychotherapeutic content more “bite-sized” may encourage better adoption of digital interventions among new mothers. Mothers also provided valuable feedback regarding the features of the MamaLift Plus device. They indicated a strong preference against voice journaling, noting that they often engage with the MamaLift Plus app while doing other activities. A majority of mothers suggest that the appropriate level of engagement with a care provider *via* the app would be at least weekly and the preferred mode of communication is text messaging.

Analysis of the user behavior is consistent with feedback collected through the feasibility questionnaire. Respondents to the feasibility questionnaire suggested that it would be preferable to use this digital tool several times per week rather than every day. This is consistent with the analysis of user behavior described above. However, the majority of participants completed all 14 lessons within the expected 3-week timeframe. This finding suggests that future versions of the MamaLift Plus mobile application offer sufficient time to enable users to “catch up” on lessons missed in the prior days if needed. Also, it is important to note that while journey entry was not an explicitly requirement of the digital therapeutic experience, non-usage of the feature will inform future technical enhancements that may promote usability and user engagement.

Moreover, MamaLift Plus has a very good safety use profile. There were no Adverse or Serious Adverse Events reported during this Human Factors Trial, in which these reporting may have been minimized by excluding potential participants from the study who answered “1/Hardly Ever” to the self-harm question on the EPDS.

The results of Human Factor Trial support the ACOG recommendations that targeted and early behavioral health interventions are needed to support the treatment and management of perinatal patients who have PPD. This is consistent with recommended guidelines from other behavioral health experts and scholars who suggest the need for early and comprehensive services especially for women at-risk.

This was a study designed for women with PPD who are interested in utilizing a digital application to address the symptoms of PPD. MamaLift Plus may be an acceptable digital tool that can be used t address the behavioral health concerns of women with mild to moderate PPD. It is a convenient, easy to use solution that women in the study would recommend to others who may also be experiencing PPD.

## Limitations

This device was not evaluated in conjunction with a clinical or mental health professional for the treatment of PPD. Confirmation of the results has not been clinically validated in this study. These are participants who self-reported their condition and outcomes, thereby reflecting their opinions in the utility, likeability and reliability of the MamaLift Plus technology application. No other inferences can be made other than the ones reported in this study.

It is difficult to generalize the results of this study broadly to all women experiencing PPD due to its small sample size (*N* = 14). Although there was substantial interest from the target patient population to participate in this study, Curio made the decision to close enrollment so that interested participants would have the opportunity to enroll in a subsequent study with a greater duration and sample size. In addition, data relative to the degree to which each therapeutic assignment was completed were not assessed in this study. Device usage data regarding differences in viewing rates of audio, video, and animation materials were also not collected nor were frequencies of when participant's log in and log out of the tool. However, examination of these aspects and other innovations will be the focus of future studies to support solutions in the effective treatment of postpartum depression.

## Conclusions

The results of this analysis indicate that behavioral health management using a mobile, digital technology can be an effective tool to support the treatment of PPD. MamaLift Plus can be used as a standalone therapy or in conjunction with direct medical care from a behavioral health provider. Results of the HFT suggest that although a digital therapeutic solution shows promise, a human engagement component supports the adoption of this technology. Additional investigations are underway to further develop the utility of treating patients with PPD using this technology.

## Data availability statement

The original contributions presented in the study are included in the article/supplementary material, further inquiries can be directed to the corresponding author/s.

## Ethics statement

This study involving human participants was reviewed and approved by Brany IRB. The patients/participants provided their written informed consent to participate in this study.

## Author contributions

JT provided critical feedback on this manuscript. IM contributed to the conceptualization and implementation of the research and took the lead in preparing the manuscript with support from MC and EN. MC is the lead contributor to the results, discussion, and interpretation. EN wrote the protocol and was in charge of the clinical direction. SD conceived the project and developed a theoretical framework for the research. SS lead the development and technology implementation of the MamaLift Plus digital therapeutic. SK served as principal investigator and contributor to the project. All authors discussed the results and contributed to the final manuscript. All authors contributed to the article and approved the submitted version.

## Funding

This study was funded by Curio Digital Therapeutics but performed by an independent non-profit lab.

## Conflict of interest

The authors declare that the research was conducted in the absence of any commercial or financial relationships that could be construed as a potential conflict of interest.

## Publisher's note

All claims expressed in this article are solely those of the authors and do not necessarily represent those of their affiliated organizations, or those of the publisher, the editors and the reviewers. Any product that may be evaluated in this article, or claim that may be made by its manufacturer, is not guaranteed or endorsed by the publisher.
